# Genomic prediction of crossbred performance based on purebred Landrace and Yorkshire data using a dominance model

**DOI:** 10.1186/s12711-016-0220-2

**Published:** 2016-06-08

**Authors:** Hadi Esfandyari, Piter Bijma, Mark Henryon, Ole Fredslund Christensen, Anders Christian Sørensen

**Affiliations:** Department of Molecular Biology and Genetics, Center for Quantitative Genetics and Genomics, Aarhus University, Tjele, 8830 Denmark; Animal Breeding and Genomics Centre, Wageningen University, Wageningen, The Netherlands; Danish Pig Research Centre, Seges, Axeltorv 3, 1609 Copenhagen V, Denmark; School of Animal Biology, University of Western Australia, 35 Stirling Highway, Crawley, WA 6009 Australia

## Abstract

**Background:**

In pig breeding, selection is usually carried out in purebred populations, although the final goal is to improve crossbred performance. Genomic selection can be used to select purebred parental lines for crossbred performance. Dominance is the likely genetic basis of heterosis and explicitly including dominance in the genomic selection model may be an advantage when selecting purebreds for crossbred performance. Our objectives were two-fold: (1) to compare the predictive ability of genomic prediction models with additive or additive plus dominance effects, when the validation criterion is crossbred performance; and (2) to compare the use of two pure line reference populations to a single combined reference population.

**Methods:**

We used data on litter size in the first parity from two pure pig lines (Landrace and Yorkshire) and their reciprocal crosses. Training was performed (1) separately on pure Landrace (2085) and Yorkshire (2145) sows and (2) the two combined pure lines (4230), which were genotyped for 38 k single nucleotide polymorphisms (SNPs). Prediction accuracy was measured as the correlation between genomic estimated breeding values (GEBV) of pure line boars and mean corrected crossbred-progeny performance, divided by the average accuracy of mean-progeny performance. We evaluated a model with additive effects only (MA) and a model with both additive and dominance effects (MAD). Two types of GEBV were computed: GEBV for purebred performance (GEBV) based on either the MA or MAD models, and GEBV for crossbred performance (GEBV-C) based on the MAD. GEBV-C were calculated based on SNP allele frequencies of genotyped animals in the opposite line.

**Results:**

Compared to MA, MAD improved prediction accuracy for both lines. For MAD, GEBV-C improved prediction accuracy compared to GEBV. For Landrace (Yorkshire) boars, prediction accuracies were equal to 0.11 (0.32) for GEBV based on MA, and 0.13 (0.34) and 0.14 (0.36) for GEBV and GEBV-C based on MAD, respectively. Combining animals from both lines into a single reference population yielded higher accuracies than training on each pure line separately. In conclusion, the use of a dominance model increased the accuracy of genomic predictions of crossbred performance based on purebred data.

**Electronic supplementary material:**

The online version of this article (doi:10.1186/s12711-016-0220-2) contains supplementary material, which is available to authorized users.

## Background

The effect of dominance, a non-additive genetic effect, has traditionally been ignored in genetic evaluation of livestock populations. There are three reasons for this: (1) lack of informative pedigrees, typically requiring large full-sib families for accurate estimates of dominance effects [1]; (2) litter effects are often confounded with family effects, particularly in prolific species, such as chickens and pigs; and (3) prediction of dominance effects involves complex computations that are often cumbersome [1, 2]. The recent advent of dense single nucleotide polymorphism (SNP) panels has, however, renewed interest in the prediction of non-additive genetic effects [3–7]. The availability of SNP genotypes increases the potential to estimate dominance effects because it enables us to determine which animals are heterozygous at each SNP and to predict the genotypic value of future matings [8]. Thus, dense SNP panels provide the technology that has been long needed to exploit dominance effects in genetic evaluation.

In some livestock production systems, including pigs, crossbred animals are used in commercial production to exploit heterosis and complementary effects. The aim of selective breeding programs in these systems is to maximize crossbred performance, where selection is carried out within pure lines using data from purebred animals [9]. However, traits evaluated on purebred populations are often genetically different from these same traits evaluated in crossbred animals because the genetic correlation between crossbred and purebred performance ($r_{pc}$) is usually less than 1 [10, 11]. Genetic correlations less than 1 are often caused by genotype-by-environment (G × E) interactions and non-additive (particularly dominance) genetic effects [12].

One of the challenges of implementing genomic selection in crossbreeding programs is to determine whether marker effects should be predicted from pure line or crossbred data. When non-additive genetic effects or G × E exist, purebred performance is likely to be a poor predictor of performance in crossbred descendants, which has led to suggest the use of a training dataset consisting of crossbred animals [11, 13, 14]. Training on crossbred data is expected to account for genetic differences between purebred and crossbred animals and for G × E. However, in practice, crossbred information is often not available because performance records and genotypes are difficult or expensive to obtain on crossbred animals. An alternative would be to train on pure line data using a dominance model, which we hypothesized would increase the accuracy of genetic evaluation of pure lines for crossbred performance if part of the deviation of $r_{pc}$ from 1 is due to dominance [15]. Previous studies have reported improved prediction accuracies by including dominance in genomic evaluation models, but most of these used models fitted to purebred data for genetic evaluation of purebred performance [4, 5, 16]. Including dominance in models for crossbred performance would further improve prediction accuracies, since dominance is the major genetic basis of heterosis. Furthermore, dominance is expected to be one of the factors that contribute to the deviation of $r_{pc}$ from 1. Thus, we hypothesized that including dominance effects in genomic prediction models would increase the prediction accuracy of purebred animals that are selected for crossbred performance. We tested this hypothesis using two approaches. First, we compared the predictive ability of genomic prediction models with either additive, or both additive and dominance effects, when the validation criterion was crossbred performance. Second, we compared the use of two separate pure-line reference populations to the use of a single reference population that combined both pure lines.

## Methods

**Fig. 1:**
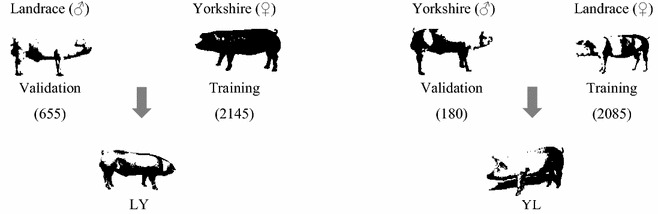
Schematic representation of the mating design. Landrace boars were mated to Yorkshire sows (and vice versa) to produce crossbred progeny. Training in both lines was on sows and validation was on boars

We used data on litter size at first parity from two pure pig lines (Landrace and Yorkshire) and their reciprocal crosses (Fig. 1). The data were supplied by the Danish Pig Research Centre (Copenhagen, Denmark).

### Purebred data

Litter sizes of 489,523 Landrace and 316,127 Yorkshire sows were used to calculate corrected phenotypic values for litter size for each line separately (see details below). Corrected phenotypic values for litter size at birth (LSc), rather than raw observations, were used as response variables for genomic prediction and to estimate additive and dominance genetic variances. The reason for using LSc as response variable was to reduce noise by removing non-genetic effects, which could be estimated much more accurately using a large dataset that includes all contemporaries and relatives, rather than using only genotyped animals. Contemporary group effects were estimated using a traditional pedigree-based linear model including herd–year–season, month at farrowing and effects of hybrid indicator (0 = pure litter and 1 = hybrid litter), age at first farrowing (linear covariate), artificial insemination (AI) (0 = natural mating and 1 = AI), along with random effects of service sire, animal additive genetic effects, and residuals. The LSc was computed as the original observations of litter size adjusted for all non-genetic effects from this model.

A total of 2740 Landrace pigs (2085 sows and 655 boars) and 2325 Yorkshire pigs (2145 sows and 180 boars) were genotyped using the Illumina PorcineSNP60 BeadChip (Illumina, San Diego, CA). Quality control of the genotype data consisted in removing SNPs with a call rate less than 90 %, SNPs with a minor allele frequency (MAF) less than 1 %, SNPs with more than 2 % missing genotypes, and SNPs that deviated strongly from Hardy–Weinberg equilibrium (P < 10^−7^). For SNPs with less than 2 % missing genotypes, the most common genotype at each SNP was identified within each population and assigned to the missing genotypes. Animals with more than 10 % missing SNP genotypes were also removed. After editing, 34,216 and 35,135 SNPs remained for 2085 Landrace and 2145 Yorkshire sows, respectively. More details about the data are in [17].

### Crossbred data

There were 7605 sows in the crossbred dataset. The crossbred animals were from the first generation of reciprocal crosses of Landrace and Yorkshire. The crossbred animals were 5575 Landrace × Yorkshire (sire–dam) and 2030 Yorkshire × Landrace (sire–dam) and were born between 2009 and 2012. Pedigrees were available for both purebred and crossbred animals and all crossbred animals could be traced back to their purebred parents. Similar to the purebred Landrace and Yorkshire data, litter size of crossbred animals were calculated by adjusting for estimates of non-genetic effects obtained from a traditional animal model with a pedigree-based relationship matrix. The model included herd-year-group, month at farrowing and a linear covariate of age at first farrowing and, as well as random animal additive genetic effects and residuals [18].

### Training and validation datasets

The purebred genotyped animals were split into training and validation datasets to evaluate the accuracy of genomic prediction for crossbred performance (Fig. 1). The Landrace training dataset consisted of 2085 sows with genotypes and LSc phenotypes. The Landrace validation dataset included 655 boars with 5575 Landrace–Yorkshire (LY) crossbred offspring. The response variable for the Landrace boars in the validation dataset was the mean LSc of their LY crossbred progeny. Thirty-two of the 655 boars in the validation dataset also had daughters (N = 320) in the Landrace training dataset. The Yorkshire training dataset included 2145 genotyped sows. Similar to Landrace, the Yorkshire validation dataset consisted of 180 genotyped boars that had 2030 daughters in the Yorkshire–Landrace (YL) crossbred dataset, and there was no direct relationship between sows of the training data and the YL crossbred animals. Relationships between sows in the training set and boars in the validation set were minimal since only three of the 180 boars had daughters (N = 30) in the Yorkshire training dataset. For genomic training on the combined pure lines, genotyped sows from both lines were combined to create a single training dataset of 4230 animals. For the combined reference population, the 30,201 SNPs that were common to the two pure lines were used.

### Linear models for genomic prediction

#### Estimation of SNP effects

Two models for genomic prediction were evaluated. The first model included only additive effects (MA) and was used to estimate the additive effect associated with each SNP:

MA $$y_{i} = \mu + \sum X_{ij} a_{j} + e_{i} ,$$

where $y_{i}$ is the phenotypic value of individual $i$ in the training data, $\mu$ is the overall mean, $X_{ij}$ is the copy number of a given allele of SNP $j$, coded 0, 1 and 2 for aa, aA and AA, respectively, $a_{j}$ is the random unknown additive effect for SNP $j$, $e_{i}$ is the residual effect for animal $i$, and Σ denotes summation over all SNPs.

The second model (MAD) included both additive and dominance effects associated with each SNP and was as follows:

MAD $$y_{i} = \mu + \sum X_{ij} a_{j} + \sum Z_{ij} d_{j} + e_{i} .$$

The definition of the elements in this model is analogous to that in model MA. In addition, $Z_{ij}$ is the indicator variable for heterozygosity of individual $i$ at SNP $j$, with $Z_{ij} = 0$ when individual $i$ is homozygous at SNP $j$ (aa or AA) and $Z_{ij} = 1$ if individual $i$ is heterozygous at SNP $j$ (aA), and $d_{j}$ is the random unknown dominance effect for SNP $j.$

The BayesC method proposed by Habier et al. [19] was used to estimate SNP effects. We used the BGLR “Bayesian general linear regression” R package developed by Perez and de los Campos [20] and its built-in default rules to set the values of hyper-parameters. A total of 100,000 iterations of the sampler were run, with the first 10,000 iterations discarded as burn-in samples. The total number of iterations and the number of burn-in samples of the chain were calculated using the *raftery.diag* function of the R package Coda [21]. Convergence of the resulting posterior distributions was assessed by the Geweke diagnostic using the Coda package [21].

#### Genomic estimated breeding values

Genomic estimated breeding values (GEBV) were calculated as the expected genotypic value of the offspring of a boar. From the estimates of additive marker effects ($\hat{a}$), the GEBV based on model MA, (${\text{GEBV}}_{\text{MA}}$) for purebred boar $i$ from breed $r$ was calculated as [22]:

1 $$\begin{aligned} GEBV_{iMA} & = \mathop \sum \limits_{j = 1}^{s} \left[\left( {S_{ij}^{1} } \right)(p_{jr} \hat{a}_{j} )\right. \\ & \quad + \left( {S_{ij}^{2} } \right)(0.5p_{jr} \hat{a}_{j} - 0.5q_{jr} \hat{a}_{j} ) \\ & \left. \quad + \left( {S_{ij}^{3} } \right)( - q_{jr} \hat{a}_{j} )\right] \\ \end{aligned}$$

where $S_{ij}^{1} , S_{ij}^{2} {\text{ and }}S_{ij}^{3}$ are indicator variables of the genotype at the *j*th SNP of the *i*th individual, with $S_{ij}^{1} = 1$ when the genotype is AA and $0$ otherwise, $S_{ij}^{2} = 1$ when the genotype is Aa or aA and 0 otherwise, and $S_{ij}^{3} = 1$ when the genotype is aa and 0 otherwise. Moreover, $p_{jr}$ and $q_{jr}$ are the frequencies of alleles A and a for the *j*th SNP in breed $r$, $\hat{a}_{j}$ is estimated additive effect of the *j*th SNP and $s$ is the total number of SNPs. Equation (1) can be reduced to the usual equation $GEBV_{MA} = \mathop \sum \nolimits_{j = 1}^{s} X_{ij} \hat{a}_{j}$, but the reason for presenting it in this way is for similarity with the equation that is given below for GEBV when dominance is included. It should be noted that the reduced equation and Eq. (1) are the same to within one constant, i.e. the correlation of GEBV based on these two equations is equal to 1 while a simple linear regression between them would result in a regression coefficient of 0.5.

With the MAD model, two types of GEBV were calculated: GEBV for purebred performance (GEBV) and GEBV for crossbred performance (GEBV-C). GEBV were calculated as the expected genotypic values of the offspring of a boar carrying a certain set of SNP genotypes, when this parent is mated at random to its own line (GEBV) or to the other pure line (GEBV-C). Thus, from the estimates of both additive ($\hat{a}$) and dominance effects ($\hat{d}$), the GEBV from model MAD for purebred boar $i$ was calculated as:

2 $$\begin{aligned} GEBV_{iMAD} & = \mathop \sum \limits_{j = 1}^{s} \left[\left( {S_{ij}^{1} } \right)(p_{jr} \hat{a}_{j} + q_{jr} \hat{d}_{j} )\right. \\ & \quad + \left( {S_{ij}^{2} } \right)(0.5p_{jr} \hat{a}_{j} + 0.5q_{jr} \hat{d}_{j} + 0.5p_{jr} \hat{d}_{j} - 0.5q_{jr} \hat{a}_{j} ) \\ & \left.\quad + \left( {S_{ij}^{3} } \right)( - q_{jr} \hat{a}_{j} + p_{jr} \hat{d}_{j} )\right] \\ \end{aligned}$$

The definition of the elements in Eq. (2) is analogous to that for ${\text{GEBV}}_{\text{MA}}$. In addition, $\hat{d}_{j}$ is the estimated dominance effect of the *j*th SNP.

For crossbred offspring, the expected genotype frequencies of the offspring of a parent depend on the allele frequency in the other pure line (denoted *ŕ* here). Thus, for animal $i$ from line $r$, the GEBV-C was calculated using Eq. 2 but with $p_{jr}$ and $q_{jr}$ replaced by *p*_*jŕ*_ and *q*_*jŕ*_, which are the frequencies of alleles A and a for the *j*th SNP in line $r'$. SNP allele frequencies in the other line were calculated based on SNP genotypes of genotyped sows in that line. For example, to predict GEBV-C for a Landrace boar, we used Eq. 2 with SNP allele frequencies calculated from all genotyped Yorkshire sows.

### Variance components

In addition to the additive variance computed from a pedigree-based animal model, we estimated genomic additive and dominance variances for the animals in the training set. A mixed linear model for individual breeding values $\left( u \right)$ and dominance deviations $\left( v \right)$ was used as follows:

$${\mathbf{y}} = \mu + {\mathbf{Z}}_{1} {\mathbf{u}} + {\mathbf{Z}}_{2} {\mathbf{v}} + {\mathbf{e}},$$

where $${\mathbf{y}}$$ is a vector of phenotypic values, $$\mu$$ is the overall mean, $${\mathbf{Z}}_{1}$$ and $${\mathbf{Z}}_{2}$$ are design matrices relating animals to their breeding values and dominance deviations, $${\mathbf{u}}$$ is a vector of breeding values, $${\mathbf{v}}$$ is a vector of dominance deviations of animals, and $${\mathbf{e}}$$ is a vector of residuals. $${\text{V}}\left( {\mathbf{u}} \right) = {\mathbf{G}}\upsigma_{\text{A}}^{2}$$, where $${\mathbf{G}}$$ is the genomic relationship matrix, which was calculated using the approach of VanRaden [23]: $${\mathbf{G}} = \frac{{{\mathbf{W}}_{{\mathbf{a}}} {\mathbf{W}}_{{\mathbf{a}}}^{ '} }}{{2\mathop \sum \nolimits_{{{\text{k}} = 1}}^{\text{m}} {\text{p}}_{\text{k}} {\text{q}}_{\text{k}} }}$$, where matrix $${\mathbf{W}}$$ has dimensions equal to the number of individuals ($$n$$) by the number of loci ($$m$$), with elements that are equal to $$2 - 2p_{k}$$ and $$- 2p_{k}$$ for opposite homozygous and $$1 - 2p_{k}$$ for heterozygous genotypes, $$p_{k}$$ is the minor allele frequency at locus $${\text{k}}$$, and $$q_{k} = 1 - p_{k}$$. The covariance matrix of dominance effects is $${\text{V}}\left( {\mathbf{v}} \right) = {\mathbf{D}}\upsigma_{\text{D}}^{2} ,$$ where $${\mathbf{D}}$$ is the genomic dominance relationship matrix and $$\sigma_{D}^{2}$$ is the dominance variance. Matrix $${\mathbf{D}}$$ was calculated as $${\mathbf{D}} = \frac{{{\mathbf{W}}_{{\mathbf{d}}} {\mathbf{W}}_{{\mathbf{d}}}^{ '} }}{{4\mathop \sum \nolimits_{{{\text{k}} = 1}}^{\text{m}} {\text{p}}_{\text{k}}^{2} {\text{q}}_{\text{k}}^{2} }}$$, where $${\mathbf{W}}_{{\mathbf{d}}}$$ has dimensions equal to the number of individuals ($$n$$) by the number of loci ($$m$$), with elements that are equal to $$- 2q_{k}^{2}$$ for genotype AA, $$2p_{k}$$$$q_{k}$$ for genotype Aa and $$- 2p_{k}^{2}$$ for genotype aa. Estimation of additive and dominance variances using these parameterizations, which match with classical quantitative genetics theory [22], were carried out using the average information restricted maximum likelihood algorithm [24] implemented in the GVCBLUP package [25].

### Validation of models

Goodness of fit for each model was evaluated by the deviance information criterion (DIC) value in the training dataset. The superiority of MAD over MA was tested by a likelihood ratio test.

Predictive ability of a model (with respect to accuracy and unbiasedness) was evaluated by comparing the GEBV of the boars in the validation dataset with the mean corrected phenotypes of their crossbred offspring. Unbiasedness of GEBV was assessed by regressing mean corrected phenotypes of crossbreds on the GEBV of the boars in both lines. A necessary condition for unbiased predictions is that the regression coefficient does not deviate significantly from 1. Prediction accuracy of GEBV was measured as the correlation between GEBV of boars in the pure lines and mean corrected crossbred-progeny performance. This correlation was divided by the average accuracy of mean-progeny performance, i.e. the mean of $\sqrt {\frac{n}{n + k},}$ where $n$ is number of daughters for each boar and $k = \left( {4 - h^{2} } \right)/h^{2}$ [2]. Here, the heritability $h^{2}$ was the narrow-sense heritability estimated from the pedigree-based linear model.

## Results

### Prediction of GEBV

MAD had better predictive ability than MA for both the Landrace and Yorkshire lines (Table 1). Including dominance in the model improved prediction accuracy of GEBV by 18 % for the Landrace line and by 5 % for the Yorkshire line. Within MAD, prediction of crossbred performance based on GEBV-C was more accurate than that based on GEBV for both lines and predictions were more accurate for the Yorkshire line than for the Landrace line (Table 1).

**Table 1:** Prediction accuracies for Landrace and Yorkshire boars based on a genomic model with only additive effects (MA) and a model with additive and dominance effects (MAD)

	Purebred^a^			Combined^b^		
	MA	MAD		MA	MAD	
	GEBV	GEBV	GEBV-C	GEBV	GEBV	GEBV-C
Landrace	0.114 (0.03)	0.135 (0.03)	0.144 (0.03)	0.167 (0.03)	0.179 (0.03)	0.207 (0.03)
Yorkshire	0.320 (0.06)	0.339 (0.06)	0.358 (0.06)	0.391 (0.06)	0.402 (0.06)	0.426 (0.06)

For both models, the validation criterion was crossbred performance

*MA* additive model, *MAD* dominance model, *GEBV* genomic estimated breeding value for purebred performance, *GEBV-C* genomic estimated breeding value for crossbred performance

^a^Purebred: training in pure lines was done separately

^b^Combined: genotyped sows from both pure lines were combined together to create a single training population

Enlarging the training dataset by combining Landrace and Yorkshire animals into a single training population increased the prediction accuracy of MA and MAD models for both lines (Table 1) with the highest increase found for the Landrace line, i.e. 33 to 46 %, whereas for the Yorkshire line, prediction accuracies increased by 19 to 22 %.

### Goodness-of-fit of models

The MAD model improved data fit over the MA model for both the Landrace and the Yorkshire data (Table 2). A lower DIC value was obtained with MAD than with MA for both lines. Measures of goodness-of-fit based on likelihood-ratio test also showed superiority of MAD over MA in fitting the data. However, this superiority was not statistically significant.

**Table 2:** The deviance information criterion (DIC), $\upchi^{2}$ value and the corresponding P value of the likelihood ratio

	MA	MAD	$\chi^{2}$ values	P value
	DIC	DIC		
Landrace	11,230.35	11,227.60	2.17	0.14
Yorkshire	11,131.54	11,121.42	2.18	0.13

*MA* additive model, *MAD* dominance model

### Bias of genomic prediction

Coefficients of regression of corrected phenotypes of crossbreds on the predicted breeding values of boars in both lines show that, for the Landrace line, the variance of the GEBV was overestimated, i.e. most regression coefficients were less than 1.0 (Table 3). When training was on the combined dataset, regression coefficients were closer to 1, which suggests that joining two lines into a single reference population reduced the bias of genomic predictions, especially for the MA model.

**Table 3:** Regression coefficients (±standard errors) of corrected litter size of crossbreds on genomic estimated breeding value for the boars in the validation dataset

	Purebred^a^			Combined^b^		
	MA	MAD		MA	MAD	
	GEBV	GEBV	GEBV-C	GEBV	GEBV	GEBV-C
Landrace	0.44 ± 0.11	0.60 ± 0.14	0.73 ± 0.17	0.71 ± 0.13	0.87 ± 0.16	1.26 ± 0.21
Yorkshire	0.69 ± 0.09	1.14 ± 0.20	1.36 ± 0.28	0.94 ± 0.18	1.24 ± 0.24	1.60 ± 0.27

For both models, the validation criterion was crossbred performance

*MA* additive model, *MAD* dominance model, *GEBV* genomic estimated breeding value for purebred performance, *GEBV-C* genomic estimated breeding value for crossbred performance

^a^Purebred: training in pure lines was done separately

^b^Combined: genotyped sows from both pure lines were combined together to create a single training population

### Estimation of variance components

Estimates of additive genetic variance and heritability obtained with the pedigree-based model differed from those obtained with the genomic models (Table 4). The estimated pedigree-based heritability was higher for the Landrace than for the Yorkshire line, whereas the genomic-based estimate of narrow sense heritability was similar for both lines. Dominance genetic variance computed using genomic was 15 and 18 % as large as the estimate of additive variance for the Landrace and Yorkshire lines, respectively.

**Table 4:** Estimates of additive genetic variance ($\upsigma_{\text{a}}^{2}$), dominance variance ($\upsigma_{\text{d}}^{2}$), and the proportions of these variances (${\text{h}}_{\text{a}}^{ 2} \;{\text{and}}\;{\text{h}}_{\text{d}}^{ 2}$) relative to phenotypic variance

Parameters	Landrace		Yorkshire	
	Pedigree	Genomic	Pedigree	Genomic
$\upsigma_{\text{a}}^{2}$	1.29 (0.03)	0.78 (0.13)	1.00 (0.03)	0.66 (0.12)
$\upsigma_{\text{d}}^{2}$	–	0.12 (0.07)	–	0.12 (0.06)
${\text{h}}_{\text{a}}^{2}$	0.10 (0.002)	0.05 (0.02)	0.08 (0.003)	0.05 (0.02)
${\text{h}}_{\text{d}}^{2}$	–	0.007 (0.01)	–	0.01 (0.01)

## Discussion

We tested whether including dominance effects in genomic prediction models increased the prediction accuracy of purebred animals for crossbred performance. We provide evidence that supports this premise by showing that including dominance increased the prediction accuracy for litter size for both the Landrace and Yorkshire lines. We also found that combining the data from the two lines into a single reference population improved prediction accuracy for both lines. Therefore, a dominance model can be used to increase the accuracy of genomic predictions for crossbred performance.

### Comparison of models

We showed that crossbred performance was predicted more accurately and with less bias by including dominance in genomic models, although only small amounts of dominance variance were detected in both the Landrace and Yorkshire lines. A possible explanation for higher prediction accuracy in spite of the small amount of dominance variance may be that the dominance variances have been underestimated. Also, in our study, the validation criterion was crossbred performance. To determine whether inclusion of dominance also increased the prediction accuracy of purebred performance, we compared the additive and dominance models by training and validation based on the performance of sows within each pure line, using five-fold cross-validation. For each line, sows with both phenotype and genotype data were split randomly into five sets. In each run, four sets were used for training and the remaining set was used for validation. The results showed that including dominance in the genetic model improved prediction accuracy also within pure lines; prediction accuracy for sow performance was improved by 11 and 9 % for the Landrace and Yorkshire lines, respectively, by including dominance (see Additional file 1). Thus, although we detected only small amounts of dominance variance, including dominance effects was beneficial both for prediction of purebred and crossbred performance.

Improvements in genomic prediction for purebred performance by including dominance effects in the genetic evaluation model were previously reported for several livestock species, including pigs, dairy cattle and sheep [3–5]. To our knowledge, no study on real data has compared additive and dominance models for crossbred performance. However, in a simulation study, Zeng et al. [13] compared additive and dominance models for genomic selection of purebred animals for crossbred performance and showed that, in the presence of dominance effects, genomic selection based on a dominance model resulted in greater improvements in crossbred performance through purebred selection than an additive model.

#### GEBV versus GEBV-C

For the MAD model, prediction accuracy of GEBV-C for crossbred performance was higher than that of GEBV, for both lines. Note that the GEBV and GEBV-C were calculated for the same boars but using different allele frequencies. GEBV is an estimated breeding value for purebred performance while GEBV-C is an estimated breeding value for crossbred performance. GEBV can be used as a selection criterion for genetic improvement within a pure line, while GEBV-C is a selection criterion to improve crossbred performance. Because the validation criterion in this study was crossbred performance, it was not surprising that GEBV-C outperformed GEBV. However, the reason for presenting GEBV was to show that if within-line selection is on crossbred performance based on GEBV rather than on GEBV-C, some loss of genetic improvement for crossbred performance is expected. We identified the top 50 Landrace and top 50 Yorkshire boars based on both GEBV and GEBV-C, and found that 42 (Landrace) and 43 (Yorkshire) boars were in the top 50 for both. This indicates that ranking of boars would be different for purebred and crossbred performance and, therefore, breeding for GEBV-C is expected to result in greater progress in performance of crossbred animals.

For both lines, we found that GEBV-C had better predictive ability than GEBV based on the dominance model. The improvement in prediction accuracy based on GEBV-C was achieved by including dominance effects in the model and using allele frequencies of the opposite line when computing GEBV-C. Dekkers et al. [26] also showed that for a two-way cross, the allele substitution effects for quantitative trait loci (QTL) or markers in one parental breed depend on the allele frequencies in the other parental breed. Thus, not using the appropriate allele frequencies to estimate breeding values can reduce responses to selection. Thus, additive models cannot maximize the genetic improvement in crossbred animals because the GEBV of an animal is the same for purebred and crossbred performance when training is on purebred data. In dominance models, predicted allele substitution effects and estimated breeding values depend on allele frequencies. A dominance model provides estimates of both additive and dominance effects and, therefore, enables the computation of allele-substitution effects using appropriate allele frequencies. The superiority in prediction accuracy of crossbred performance based on GEBV-C over GEBV that we found here is in agreement with findings of Esfandyari et al. [15].

To estimate GEBV-C for purebred boars, we used SNP allele frequencies on the genotyped sows of the opposite line. However, a more accurate approach would be to use SNP allele frequencies for the selection candidates of the opposite line. For instance, to estimate GEBV-C for Landrace boars, SNP allele frequencies could be calculated on the 2450 Yorkshire sows that were mated to the Landrace boars to produce the crossbred progeny. However, since these sows were not genotyped, we used SNP allele frequencies for the sows of the relevant generation, which correspond to SNP allele frequencies estimated for the selection candidates.

#### BayesC versus GBLUP

In this study, we used the BayesC method to estimate the additive and dominance effects of SNPs [for the distribution of estimated effects (see Additional file 2)]. These estimated effects were then used to calculate GEBV based on the MA and MAD models. We also compared the predictive ability of the MA and MAD models to that of genomic best linear unbiased prediction (GBLUP). For this purpose, we used the estimates of additive and dominance effects from GBLUP to calculate the GEBV for the boars of both pure lines. Estimates of additive and dominance effects of each SNP for GBLUP were derived by back-solving the estimated breeding values and dominance deviations of the animals used for training [27]. Using the MAD model for GBLUP also resulted in greater predictive ability than using the MA model. Also, with GBLUP, prediction of crossbred performance based on GEBV-C was more accurate than that based on GEBV in both lines (see Additional file 3).

### Difference in prediction accuracy between Landrace and Yorkshire

Our results showed that prediction accuracy for crossbred performance in Yorkshire boars was higher than in Landrace boars with both the MAD and MA models, although the training populations and heritabilities of the two lines were similar. The pedigree-based prediction accuracy was also higher for the Yorkshire boars than for the Landrace boars (results not shown). To investigate the possible reasons for these differences, we carried out additional analyses. First, we compared the within-line prediction accuracy based on the MA and MAD models for each line by using sows for training and validation. The mean prediction accuracies for within-line performance of sows were equal to 0.15 and 0.22 for the Landrace and Yorkshire lines, respectively. The higher prediction accuracy for sow performance of Yorkshire sows may explain the higher prediction accuracy for crossbred performance for this line compared to the Landrace line. Second, we compared the variance of the off-diagonal elements of the genomic relationship matrix of training animals for the two lines and found these to be very small for both lines but larger for Yorkshire (0.0069) than for Landrace (0.0031) animals. This larger variation in genomic relationships may also explain the greater prediction accuracy for Yorkshire animals compared to Landrace animals. Third, we compared the average genomic relationship between boars and sows of the same line and found a higher relationship between boars and sows for the Yorkshire line (0.0014) than for the Landrace line (~0). These three reasons could all contribute to the higher observed prediction accuracy for the Yorkshire than for the Landrace boars.

### Benefits of using a combined reference population for genomic prediction

Combining animals from pure lines into a single training set improved prediction accuracy for both Landrace and Yorkshire lines for all models. Combining populations into a common reference dataset is often argued to be an obvious way of increasing GEBV accuracies [28, 29]. However, the increase in accuracy of GEBV found by combining populations depends on the relatedness between the populations and the consistency of the linkage disequilibrium (LD) between SNPs and QTL [30]. Using the same dataset as used here, Wang et al. [31] examined the consistency of LD between the Landrace and Yorkshire breeds. For SNPs with a pairwise distance less than 10 kb, they found a relatively high correlation of LD phase that was equal to 0.77. This may explain the improvement in prediction accuracy obtained by combining Landrace and Yorkshire animals into a single training population in our study. A high correlation of LD phase between the Landrace and Yorkshire breeds was also reported by Badke et al. [32]. Furthermore, there is a fundamental difference between this study and previous studies that reported increases in accuracy of prediction when combining populations into a single reference, i.e. in our study, validation was on crossbred performance of the two lines, whereas in other studies, validation was on pure line performance of either line. Validation on crossbred rather than purebred performance may also explain the improvement in prediction accuracy obtained in our study when combining Yorkshire and Landrace populations. In a simulation study, Esfandyari et al. [15] showed that, when the correlation of LD phase between two breeds is high, accuracy of GEBV for crossbred performance is increased if animals from the two breeds are combined into a single reference population to estimate SNP effects.

### Additive and dominance genetic variances of litter size

We used a breeding (or classical) model rather than a genotypic model to estimate additive and dominance variances, similar to Vitezica et al. [33]. The breeding model partitions the genotypic value at a SNP into an additive breeding value and dominance deviation. Resulting estimates of variances of breeding values and dominance deviations are comparable with pedigree-based estimates. The genotypic model partitions genetic variance into additive and dominance variances a manner that does not enable direct comparison to pedigree-based estimates, i.e. the additive variance is the variance of additive effects. The difference between these two models is discussed in [33].

For both lines, estimates of additive variance based on pedigree data differed from those based on genomic information, probably because animals used to estimate genomic variances represented a small proportion of all the animals. Based on the present data, the estimated dominance variance was 15 and 18 % as large as the estimate of additive variance for the Landrace and Yorkshire lines, respectively. In pigs, significant contributions of dominance genetic variance have been reported. Lopes et al. [6] found ratios of 13 and 21 % for number of teats and back fat, respectively, in the Landrace breed when using the genotypic model. However, these values decreased to 0.08 and 0.16 % for number of teats and back fat, respectively, when using a breeding model. Vitezica et al. [33] argued that the genotypic model overestimates the dominance genetic variance and, consequently, underestimates additive genetic variance. Su et al. [5] showed that the estimated dominance variance was 26 % as large as the additive variance for daily gain in Danish Duroc pigs. However, since they used the genotypic model, the reported variance for dominance was overestimated. Based on pedigree estimates, Culbertson et al. [34] reported that the ratio of dominance to additive variances for different traits in pigs ranged from 11 to 78 %. These results indicate that dominance variance is important for complex traits in pigs.

## Conclusions

Dominance models resulted in higher prediction accuracy of crossbred performance for purebred animals than additive models. This is probably because the dominance model accounts for part of the deviation from 1 of the genetic correlation between crossbred and purebred performance in crossbreeding programs. Furthermore, we found that combining animals from the two lines into a single reference population improved prediction accuracy in the two lines.

## Additional files

10.1186/s12711-016-0220-2 Within-line prediction accuracy. Results for within-line prediction accuracy for Landrace and Yorkshire sows under two genomic models.
10.1186/s12711-016-0220-2 Distribution of estimated additive and dominance effects of SNPs. Distribution of estimated additive and dominance effects of SNPs for Landrace and Yorkshire lines.
10.1186/s12711-016-0220-2 Predictive ability of MA and MAD based on GBLUP. Prediction accuracy for Landrace and Yorkshire boars under two genomic models.

## References

[1] Misztal I, Varona L, Culbertson M, Gengler N, Betrand JK, Mabry J (1998). Studies on the value of incorporating the effect of dominance in genetic evaluations of dairy cattle, beef cattle and swine. Biotechnol Agron Soc Environ.

[2] Mrode RA, Thompson R (2005). Linear models for the prediction of animal breeding values.

[3] Moghaddar N, Swan AA, van der Werf JHJ (2014). Comparing genomic prediction accuracy from purebred, crossbred and combined purebred and crossbred reference populations in sheep. Genet Sel Evol.

[4] Sun C, VanRaden PM, Cole JB, O’Connell JR (2014). Improvement of prediction ability for genomic selection of dairy cattle by including dominance effects. PLoS One.

[5] Su G, Christensen OF, Ostersen T, Henryon M, Lund MS (2012). Estimating additive and non-additive genetic variances and predicting genetic merits using genome-wide dense single nucleotide polymorphism markers. PLoS One.

[6] Lopes MS, Bastiaansen JWM, Janss L, Bovenhuis H, Knol EF. Using SNP markers to estimate additive, dominance and imprinting genetic variance. In: Proceedings of the 10th world congress on genetics applied to livestock production: 17–22 August 2014; Vancouver; 2014. https://asas.org/docs/default-source/wcgalp-proceedings-oral/220_paper_9459_manuscript_651_0.pdf?sfvrsn=2.

[7] Wittenburg D, Melzer N, Reinsch N (2015). Genomic additive and dominance variance of milk performance traits. J Anim Breed Genet.

[8] Toro MA, Varona L (2010). A note on mate allocation for dominance handling in genomic selection. Genet Sel Evol.

[9] Wei M, van der Steen HAM (1991). Comparison of reciprocal recurrent selection with pure-line selection systems in animal breeding (a review). Anim Breed Abstr.

[10] Wei M, van der Werf JHJ (1994). Maximizing genetic response in crossbreds using both purebred and crossbred information. Anim Prod.

[11] Dekkers JCM (2007). Marker-assisted selection for commercial crossbred performance. J Anim Sci.

[12] van der Wei M, Werf JHJ, Brascamp EW (1991). Relationship between purebred and crossbred parameters. 2. Genetic correlation between purebred and crossbred performance under the model with 2 loci. J Anim Breed Genet.

[13] Zeng J, Toosi A, Fernando RL, Dekkers JCM, Garrick DJ (2013). Genomic selection of purebred animals for crossbred performance in the presence of dominant gene action. Genet Sel Evol.

[14] Esfandyari H, Sorensen AC, Bijma P (2015). A crossbred reference population can improve the response to genomic selection for crossbred performance. Genet Sel Evol.

[15] Esfandyari H, Sorensen AC, Bijma P (2015). Maximizing crossbred performance through purebred genomic selection. Genet Sel Evol.

[16] Ertl J, Legarra A, Vitezica ZG, Varona L, Edel C, Emmerling R (2014). Genomic analysis of dominance effects on milk production and conformation traits in Fleckvieh cattle. Genet Sel Evol.

[17] Guo X, Christensen OF, Ostersen T, Wang Y, Lund MS, Su G (2015). Improving genetic evaluation of litter size and piglet mortality for both genotyped and nongenotyped individuals using a single-step method. J Anim Sci.

[18] Nielsen B, Velander I, Ostersen T, Henryon M, Christensen OF. Nurse capacity in crossbred sows and genetic correlation to purebred fertility. In: Proceedings of the 10th world congress on genetics applied to livestock production: 17–22 August 2014; Vancouver; 2014. https://asas.org/docs/default-source/wcgalp-proceedings-oral/368_paper_8899_manuscript_295_0.pdf?sfvrsn=2.

[19] Habier D, Fernando RL, Kizilkaya K, Garrick DJ (2011). Extension of the Bayesian alphabet for genomic selection. BMC Bioinformatics.

[20] Perez P, de los Campos G (2014). Genome-wide regression and prediction with the BGLR statistical package. Genetics..

[21] Plummer M, Best N, Cowles K, Vines K (2006). CODA: convergence diagnosis and output analysis for MCMC. R News.

[22] Falconer DS, Mackay TFC (1996). Introduction to quantitative genetics.

[23] VanRaden PM (2008). Efficient methods to compute genomic predictions. J Dairy Sci.

[24] Gilmour AR, Thompson R, Cullis BR (1995). Average information REML: an efficient algorithm for variance parameter estimation in linear mixed models. Biometrics.

[25] Wang CK, Prakapenka D, Wang SW, Pulugurta S, Runesha HB, Da Y (2014). GVCBLUP: a computer package for genomic prediction and variance component estimation of additive and dominance effects. BMC Bioinform.

[26] Dekkers JCM (1999). Breeding values for identified quantitative trait loci under selection. Genet Sel Evol.

[27] Stranden I, Garrick DJ (2009). Technical note: derivation of equivalent computing algorithms for genomic predictions and reliabilities of animal merit. J Dairy Sci.

[28] Lund MS, Su G, Janss L, Guldbrandtsen B, Brøndum RF (2014). Invited review: genomic evaluation of cattle in a multi-breed context. Livest Sci.

[29] de Roos APW, Hayes BJ, Goddard ME (2009). Reliability of genomic predictions across multiple populations. Genetics.

[30] Daetwyler HD, Kemper KE, van der Werf JHJ, Hayes BJ (2012). Components of the accuracy of genomic prediction in a multi-breed sheep population. J Anim Sci.

[31] Wang L, Sorensen P, Janss L, Ostersen T, Edwards D (2013). Genome-wide and local pattern of linkage disequilibrium and persistence of phase for 3 Danish pig breeds. BMC Genet.

[32] Badke YM, Bates RO, Ernst CW, Schwab C, Steibel JP (2012). Estimation of linkage disequilibrium in four US pig breeds. BMC Genomics.

[33] Vitezica ZG, Varona L, Legarra A (2013). On the additive and dominant variance and covariance of individuals within the genomic selection scope. Genetics.

[34] Culbertson MS, Mabry JW, Misztal I, Gengler N, Bertrand JK, Varona L (1998). Estimation of dominance variance in purebred Yorkshire swine. J Anim Sci.

