# Supplementary material for: Genomic prediction of crossbred performance based on purebred Landrace and Yorkshire data using a dominance model
Source: Genet Sel Evol. 2016 Jun 8;48:40. doi: 10.1186/s12711-016-0220-2 (PMC4899891; doi:10.1186/s12711-016-0220-2)
Supplement: Supplementary file 1 — 10.1186/s12711-016-0220-2 Within-line prediction accuracy. Results for within-line prediction accuracy for Landrace and Yorkshire sows under two genomic models. [file 12711_2016_220_MOESM1_ESM.docx]

**Additional file 1:**

**Within line prediction accuracy.**

Prediction of sow performance by training on sows within each line.

| Within line prediction accuracy for sows of Landrace and Yorkshire under two genomic model | | |
| --- | --- | --- |
|  | **Purebred** | |
|  | **MA** | **MAD** |
| Landrace | 0.156 | 0.173 |
| Yorkshire | 0.222 | 0.242 |
| MA: additive model  MAD: dominance model  For both models validation criterion was purebred performance. | | |
