# Supplementary material for: Genomic prediction of crossbred performance based on purebred Landrace and Yorkshire data using a dominance model
Source: Genet Sel Evol. 2016 Jun 8;48:40. doi: 10.1186/s12711-016-0220-2 (PMC4899891; doi:10.1186/s12711-016-0220-2)
Supplement: Supplementary file 2 — 10.1186/s12711-016-0220-2 Distribution of estimated additive and dominance effects of SNPs. Distribution of estimated additive and dominance effects of SNPs for Landrace and Yorkshire lines. [file 12711_2016_220_MOESM2_ESM.docx]

**Additional file 2:**

Distribution of estimated additive and dominance effects of SNPs for Landrace and Yorkshire.

|  |  |
| --- | --- |
