# Supplementary material for: Genomic prediction of crossbred performance based on purebred Landrace and Yorkshire data using a dominance model
Source: Genet Sel Evol. 2016 Jun 8;48:40. doi: 10.1186/s12711-016-0220-2 (PMC4899891; doi:10.1186/s12711-016-0220-2)
Supplement: Supplementary file 3 — 10.1186/s12711-016-0220-2 Predictive ability of MA and MAD based on GBLUP. Prediction accuracy for Landrace and Yorkshire boars under two genomic models. [file 12711_2016_220_MOESM3_ESM.docx]

**Additional file 3:**

**Predictive ability of MA and MAD based on the GBLUP.**

| Prediction accuracy for boars of Landrace and Yorkshire under two genomic model | | | |
| --- | --- | --- | --- |
|  | **MA** | **MAD** | |
|  | **GEBV** | **GEBV** | **GEBV-C** |
| Landrace | 0.106 | 0.113 | 0.142 |
| Yorkshire | 0.302 | 0.312 | 0.326 |
| MA: additive model  MAD: dominance model  GEBV: genomic estimated breeding value for purebred performance.  GEBV-C: genomic estimated breeding value for crossbred performance.  For both models validation criterion was crossbred performance. | | | |
